# Supplementary material for: Aluminium(III) Oxide—The Silent Killer of Bacteria
Source: Molecules. 2023 Jan 3;28(1):401. doi: 10.3390/molecules28010401 (PMC9822385; doi:10.3390/molecules28010401)
Supplement: Supplementary file 1 [file molecules-28-00401-s001.zip › molecules-2118860-supplementary.pdf]

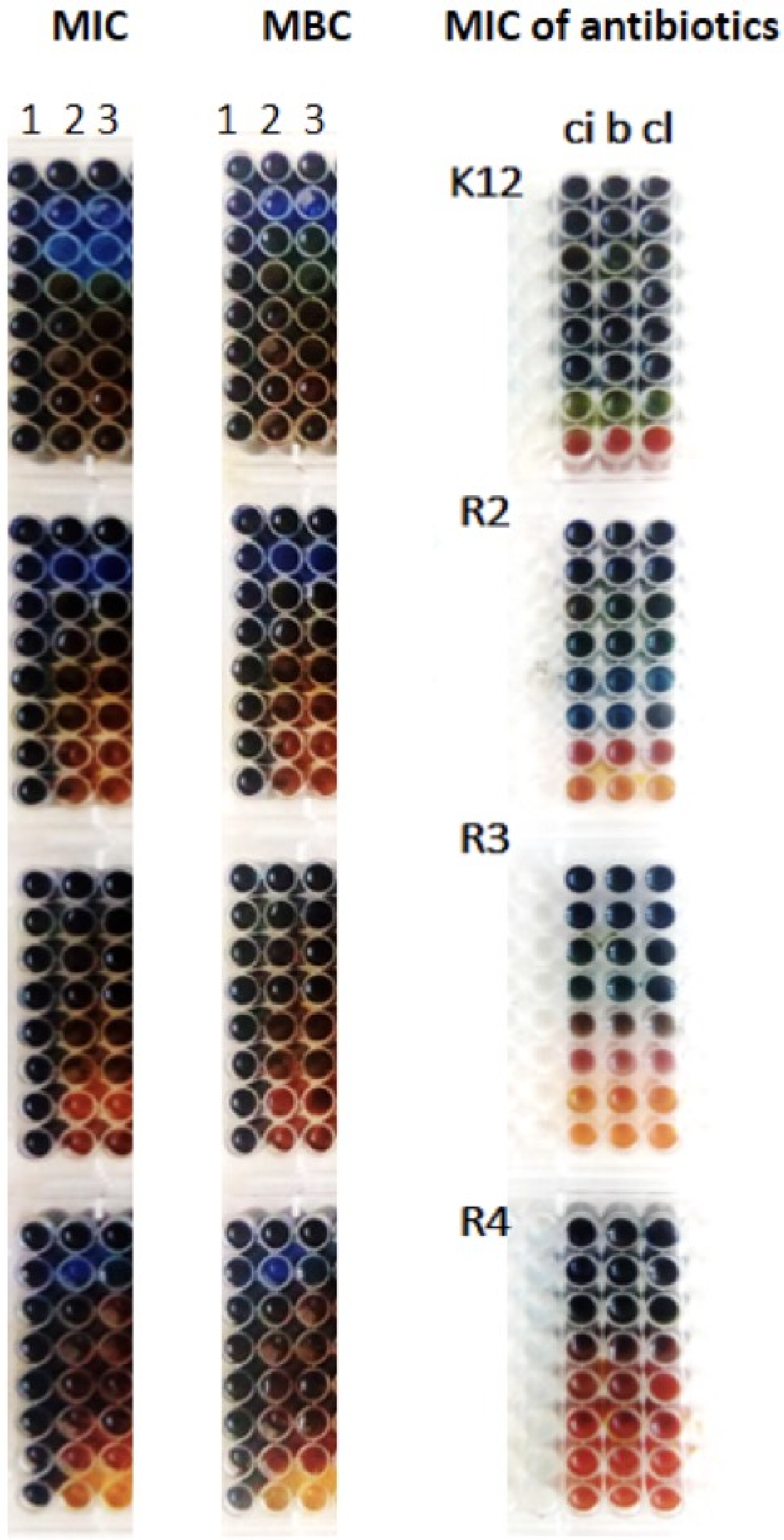

Figure S1. Examples of MIC and MBC on microplates with different concentration of studied compounds (µg/mL). The numbers correspond to sample names 1 = AAO Clean, 2 = AAO Cl , 3 = AAO Cl+Cr. Resazurin was added as an indicator of microbial growth with K12, R2, R3, and R4 strains with tested AAO matrices (for which the statistical analysis shown in Table 1). Additionally, examples of MIC with different strains K12, R2, R3, and R4 of studied antibiotics with ciprofloxacin (ci), bleomycin (b), and cloxacillin (cl) in (µg/mL).

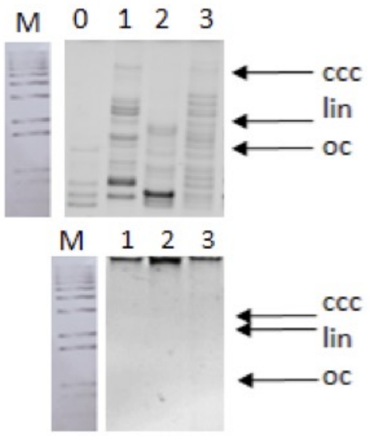

Figure S2. An example of an agarose gel electrophoresis separation of isolated plasmids DNA from selected R4 *E. coli* strains reacted with different AAO membranes (upper picture). 0 = normal plasmid not treated with AAO membranes, 1 = plasmid treated with clean AAO Clean, 2 = plasmid treated with AAO Cl, 3 = plasmid treated with AAO Cl+Cr, as shown in Figure 4, and digested with repair Fpg protein (lower picture). M = marker. 1 = plasmid treated with clean AAO Clean, 2 = plasmid treated with AAO Cl, 3 = plasmid treated with AAO Cl+Cr.

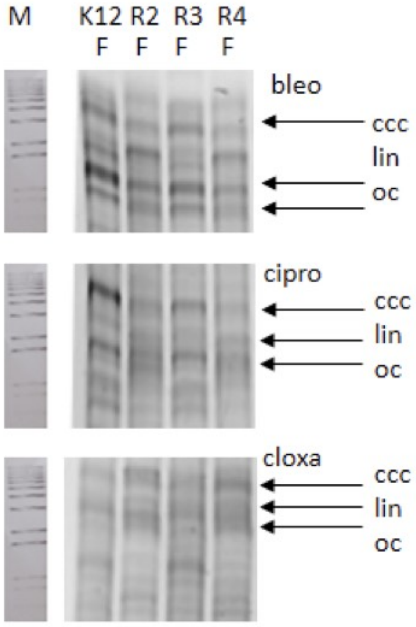

Figure S3. Example of an agarose gel electrophoresis separation of isolated plasmids DNA from K12 and R4 strains modified with antibiotics: bleomycin, ciprofloxacin, and cloxacillin digested (or not) with repair enzymes Fpg. M = marker.
